# A scoping review of the literature on the prevalence and correlates of anxiety and depression among undergraduate health science students

**DOI:** 10.1192/j.eurpsy.2024.627

**Published:** 2024-08-27

**Authors:** G. Agyapong-Opoku, B. Agyapong, G. Obuobi-Donkor, E. Eboreime

**Affiliations:** ^1^School of Health and Health Performance, Dalhousie University, Halifax; ^2^Department of Psychiatry, University of Alberta, Edmonton; ^3^Department of Psychiatry, Dalhousie University, Halifax, Canada

## Abstract

**Introduction:**

Health science students in post-secondary institutions experience high levels of depression and anxiety due to increased stress levels, workload, low socioeconomic status, and history of family mental illness, among other factors. Given the significant negative impact that depression and anxiety can have on undergraduate health science students, it is essential to understand the prevalence and correlation of these conditions in this population.

**Objectives:**

This scoping review aims to identify, document and analyze the literature on the prevalence and determinants of anxiety and depression among undergraduate health sciences students and identify gaps in knowledge for future research.

**Methods:**

The scoping review was planned and executed by the Preferred Reporting Items for Systematic Reviews and Meta-Analyses extension for the Scoping Reviews statement. A comprehensive and systematic search was carried out for five databases, namely MEDLINE, Scopus, EMBASE, CINAHL and PubMed.

**Results:**

From the literature identified by our search strategy, the lowest prevalence for anxiety was 5.8%, and the highest was 82.6%, with a median of 44.25%. The prevalence of depression ranged from a high of 88.8% to a low of 2.1%, with a median value of 34.8%. Our analysis revealed that correlates of anxiety and depression among health science students include sociodemographic factors such as age, sex, gender, relationships, ethnicity and family history, personal health conditions, and academic and socioeconomic issues.

**Image:**

**Figure:**
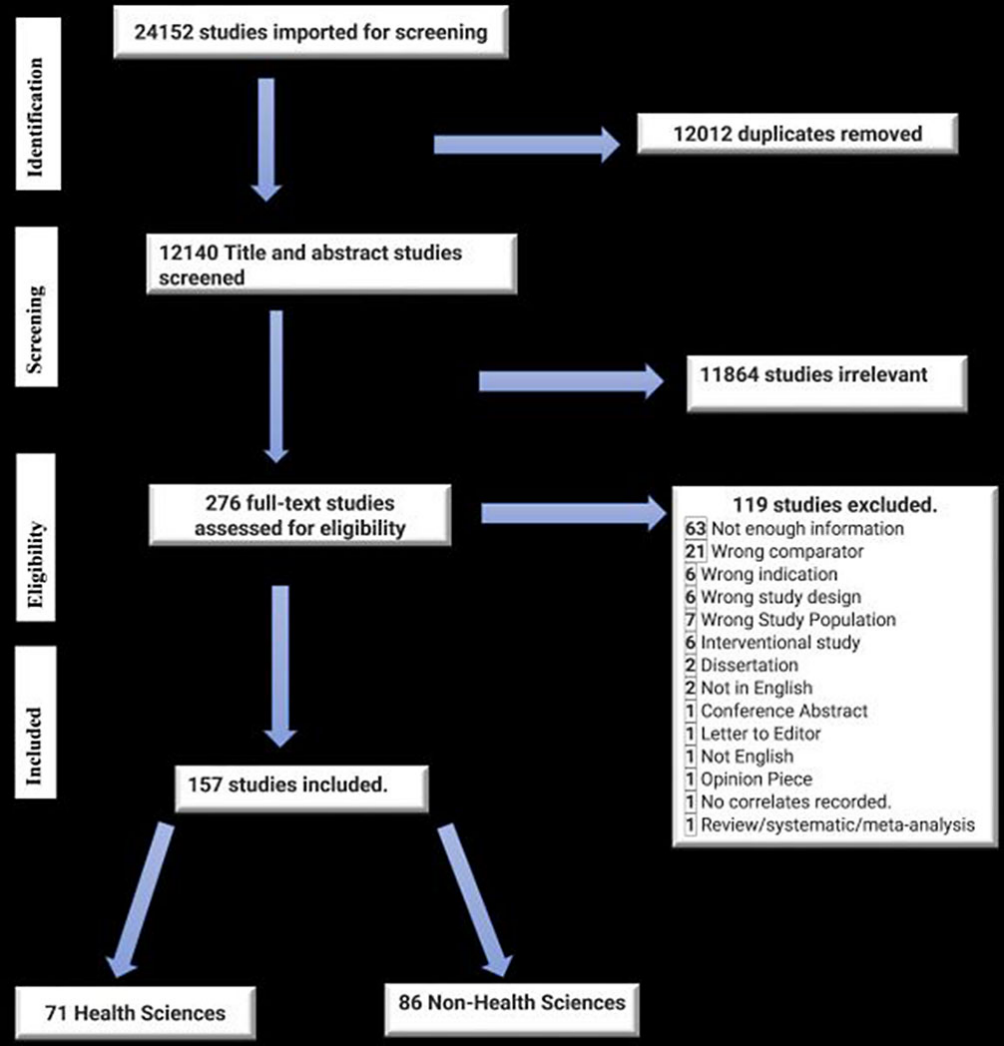

**Conclusions:**

With the high incidence of anxiety and depression among health science students, there is an increasing need to find practical remedies to support these students. It is also essential for policymakers and university authorities to implement interventions such as supportive text messages and other strategies geared toward providing support and improving the psychological well-being of health science students.

**Disclosure of Interest:**

None Declared

